# Supplementary material for: Development of a computer-aided design software for the quantitative evaluation of aesthetic damage
Source: PLoS One. 2019 Dec 18;14(12):e0226322. doi: 10.1371/journal.pone.0226322 (PMC6919621; doi:10.1371/journal.pone.0226322)
Supplement: S1 Code site — 1. (ZIP) [file pone.0226322.s001.zip › [EESC_jr][Projeto]Relatorio_07_21/reports/templates_zip/gentelella-master/vendors/Flot/examples/zooming/index.html]

Flot Examples: Selection and zooming


## Selection and zooming

Selection support makes it easy to construct flexible zooming schemes. With a few lines of code, the small overview plot to the right has been connected to the large plot. Try selecting a rectangle on either of them.

Copyright © 2007 - 2014 IOLA and Ole Laursen
